# Gastrointestinal nematodes and anthelmintic resistance in Danish goat herds☆

**DOI:** 10.1051/parasite/2014038

**Published:** 2014-07-31

**Authors:** Signe A. Holm, Camilla R. L. Sörensen, Stig M. Thamsborg, Heidi L. Enemark

**Affiliations:** 1 Section of Bacteriology, Pathology and Parasitology, National Veterinary Institute, Technical University of Denmark DK-1870 Frederiksberg C Denmark; 2 Parasitology and Aquatic Diseases, Department of Veterinary Disease Biology, University of Copenhagen Dyrlægevej 100 DK-1870 Frederiksberg C Denmark

**Keywords:** Goat, Parasites, Nematode, Parasite control, Herd management, Anthelmintic resistance

## Abstract

The prevalence of gastrointestinal parasites in Danish goats and the presence of anthelmintic resistance (AR) in 10 selected herds were investigated during April–September 2012. All Danish herds (*n* = 137) with 10 or more adult goats were invited to participate, and of these 27 herds met the inclusion criterion of more than 10 young kids never treated with anthelmintics. Questionnaire data on management were collected, and faecal samples from 252 kids were analysed by the McMaster technique. From all herds with a mean faecal egg count (FEC) above 300 eggs per g of faeces, pooled samples were stained with peanut agglutinin (PNA) for specific detection of *Haemonchus contortus*. Strongyle eggs were detected with an individual prevalence of 69%, including *Nematodirus battus* (3.6%) and other *Nematodirus* species (15.0%). *Eimeria* spp. were observed in 99.6% of the kids. *H. contortus* was found in 11 of 12 (92%) tested herds. Anthelmintics were used in 89% of the herds with mean treatment frequencies of 0.96 and 0.89 treatments per year for kids and adults, respectively. In 2011, new animals were introduced into 44% of the herds of which 25% practised quarantine anthelmintic treatments. In 10 herds the presence of AR was analysed by egg hatch assay and FEC reduction tests using ivermectin (0.3 mg/kg) or fenbendazole (10.0 mg/kg). AR against both fenbendazole and ivermectin was detected in seven herds; AR against fenbendazole in one herd, and AR against ivermectin in another herd. In conclusion, resistance to the most commonly used anthelmintics is widespread in larger goat herds throughout Denmark.

## Abstract (fr)

La prévalence des parasites gastro-intestinaux chez les chèvres du Danemark et la présence de résistance aux anthelminthiques (AR) dans 10 troupeaux sélectionnés ont été étudiées d’avril à septembre 2012. Tous les troupeaux danois (*n* = 137) de plus de 10 chèvres adultes ont été invités à participer, et de ces 27 troupeaux 10 remplissait le critère d’inclusion de plus de 10 jeunes n’ayant jamais été traités avec des anthelminthiques. Les données du questionnaire sur la gestion ont été recueillies, et des échantillons fécaux de 252 jeunes ont été analysés par la technique McMaster. De tous les troupeaux avec un nombre moyen d’œufs fécaux (FEC) au-dessus de 300 œufs par gramme de fèces, des échantillons groupés ont été colorées avec l’agglutinine d’arachide (PNA) pour la détection spécifique d’*Haemonchus contortus*. Les œufs de strongles ont été détectés avec une prévalence individuelle de 69 %, dont 3,6 % *Nematodirus battus* et 15 % d’autres espèces de *Nematodirus*. *Eimeria* spp. a été observé chez 99,6 % des jeunes. *H. contortus* a été trouvée chez 11 des 12 troupeaux testés (92 %). Les anthelminthiques ont été utilisés dans 89 % des troupeaux avec des fréquences moyennes de traitement de respectivement 0,96 et 0,89 traitement par an pour les jeunes et les adultes. En 2011, de nouveaux animaux ont été introduits dans 44 % des troupeaux dont 25 % pratiquaient des traitements anthelminthiques de quarantaine. Dans 10 troupeaux la présence d’AR a été analysé par des essais d’éclosion des œufs et des tests de réduction de la FEC à l’ivermectine (0,3 mg/kg) ou au fenbendazole (10,0 mg/kg). La résistance aux anthelminthiques à la fois au fenbendazole et à l’ivermectine a été détecté dans sept troupeaux ; l’AR au fenbendazole dans un troupeau, et l’AR à l’ivermectine dans un autre troupeau. En conclusion, la résistance aux anthelminthiques les plus couramment utilisés est très répandue dans les grands troupeaux de chèvres dans tout le Danemark.

## Introduction

Helminth infections, in particular gastrointestinal nematodes, are found worldwide and are among the most economically important diseases in goats [4, 17, 21, 35, 37]. Control of gastrointestinal nematodes is predominantly based on the use of anthelmintic drugs, but the emergence of anthelmintic resistance (AR) in trichostrongyles in the last three decades represents a major threat to the production of goats [25, 28]. In Denmark resistance against one or more of the broad-spectrum anthelmintics was first reported in Danish goats in 1996 [31], but no comprehensive surveys have been performed since then.

AR is believed to develop faster in goats than in sheep [7, 47, 51], and although nematodes are generally host-specific, goats and sheep share several species, enabling transmission of resistant nematodes from one species to the other [41, 50]. This aspect makes assessment of AR in goats important, even in countries, such as Denmark, where goats are of minor importance in livestock production. In 2012 there were 23,353 milk, meat or fibre goats in Denmark, distributed among 3,195 herds [26]. The average herd size was 7.2 goats, and 89% of the herds had 1–9 goats. Hence, goats are predominantly kept as hobby animals. *Haemonchus contortus* is a species that has repeatedly been associated with AR [3, 36, 40, 41]. Furthermore, this parasite is increasingly becoming a problem in sheep and goats in temperate areas [30, 33, 43] and has been reported as far north as the Polar Circle [14].

The objectives of the present study were to determine the prevalence of gastrointestinal parasites in Danish goats with a special focus on *H. contortus*, and to assess the occurrence of AR in selected Danish goat herds.

## Materials and methods

### Study design and selection of farms

In April 2012 an invitation to participate in the prevalence study was submitted to all Danish goat herds registered with 10 or more adult goats, according to the Central Animal Husbandry Register (*n* = 137 herds). In addition, the invitation was posted on the Danish Goat Association’s website (www.goat-dgu.dk). Twenty-seven (19.7%) herds met the inclusion criterion of more than 10 young kids never treated with anthelmintics. Questionnaire data on herd management and practices were collected from all owners (*n* = 27), who also submitted rectally obtained faecal samples from at least 10 kids for initial screening. In a subset of herds (*n* = 10) with a minimum of seven kids and a mean faecal egg count (FEC) > 150 eggs per g faeces (epg), the presence of AR against ivermectin (IVM) and fenbendazole (FBZ) was analysed using a faecal egg count reduction test (FECRT) and egg hatch assay (EHA). A single herd (#18) was examined earlier in 2012 [36].

### Sampling and laboratory analysis

#### Coprological analysis

Faecal samples were stored in individually labelled plastic bags, and submitted to the laboratory on the day of collection. Faeces was scored for consistency on a scale of 0–5 (0 = hard pellets, 1 = moist pellets, 2 = sticky, clumped pellets, 3 = soft, paste-like with no pellet structure, 4 = watery diarrhoea, 5 = watery, bloody diarrhoea), vacuum-packed and stored in the dark at room temperature until analysis within one-three days. Helminth eggs and oocysts were quantified using a modified McMaster method [22] with a sensitivity of 5 epg, and eggs were identified to species or genus level [45].

Peanut Agglutinin Staining (PNA) was performed to detect *H. contortus* eggs [16]. From all herds with a mean FEC ≥ 300 epg, the remaining faeces was pooled, and eggs were isolated, washed and stained with PNA (0.16 mg/mL) and purified by flotation in Percoll. All or a minimum of 100 strongyle eggs were recorded and the proportion of green fluorescent eggs was estimated using a fluorescence microscope (Leica DMR A 2, 10× objective, band-pass filter 450–490 nm, long-pass filter 515 nm).

#### Faecal egg count reduction test

A FECRT was performed according to the World Association for the Advancement of Veterinary Parasitology (WAAVP) guidelines [9]. On each farm kids were divided into one-three treatment groups, depending on the herd size, and treated either subcutaneously with IVM (0.3 mg/kg) (Ivomec® Vet., Merial Norden), orally with FBZ (10.0 mg/kg) (Panacur® Vet., MSD Animal Health) or were left untreated as controls. None of the drugs were registered for use in goats in Denmark but were applied at 1½ (IVM) and 2 (FBZ) times the recommended sheep dose. All animals were dosed according to individual weight by electronic scales. In farms with ≥25 kids, animals were divided into two treatment groups of 10 kids each (IVM and FBZ) and one control group of 5–10 kids. In farms with 15–24 kids the animals were divided into one treatment group of 10–12 kids (IVM) and one control group of 5–12 kids. In farms with 7–14 kids all animals were treated with IVM. Resampling was done 13–14 days posttreatment. PNA staining was performed in the pretreatment sample from the kid with the highest pretreatment FEC (minimum FEC ≥ 300 epg).

#### Egg hatch assay

An EHA for detection of BZ resistance was performed as described by Coles et al. [9] with modifications [52]. Nematode eggs were isolated from pooled pretreatment samples from each of the 10 herds. A suspension with a known egg concentration was prepared and approximately 100 eggs in 1990 μL distilled water were placed in each of 22 wells, on a 24-microwell plate. Ten μL of nine different concentrations of thiabendazole (TBZ) were added to 18 of the wells, each concentration in duplicate. The remaining four wells were used as negative and positive controls. Plates were incubated for 48 h and then one drop of Lugol’s iodine was added to each well. Wells were examined by inverse microscopy and the numbers of strongyle eggs and larvae in each well were recorded.

#### Questionnaire

All 27 participating farmers were asked to fill in a questionnaire concerning farm details (e.g. size and composition of goat flock), management practices (e.g. management of goats during kidding and grazing seasons, grazing management), drug use (e.g. choice of anthelmintics, treatment strategy) and knowledge about AR. The questionnaire was either answered by e-mail or the farmers were interviewed by telephone or face-to-face.

### Data analysis

Data from the prevalence study, FECRT and questionnaire were summarised by descriptive statistics. FECs of different groups were compared by means of analysis of variance on log(*x* + 1)-transformed counts. The FEC reduction percentage (FECR) was calculated using three different methods:

(1) $$\mathrm{FECR}=1001-T_{2}T_{1}\times C_{1}C_{2}$$

where T_1_ and T_2_ are the arithmetic means of FECs of the treatment group before and after treatment, and C_1_ and C_2_ are the arithmetic means of FECs of the control group at the same time points. According to this method resistance is indicated when FECR < 90% [39].

(2) $$\mathrm{FECR}=1001-T_{2}C_{2}$$

A 95 % confidence interval was calculated:

$$95\%\mathrm{CI}=1001-T_{2}C_{2}\;\exp\pm 2048\sqrt{Y_{2}}$$

where T_2_ and C_2_ are the arithmetic means of FECs of the treatment group and the control group after treatment. When using this method of calculation, AR is present if the FECR < 95% and the lower confidence limit is <90%. If only one of these criteria is present, AR is suspected [9].

(3) $$\mathrm{FECR}=100(1-T_{2}T_{1})$$

where T_1_ and T_2_ are the arithmetic mean FECs of the treatment group before and after treatment, respectively. This method uses a threshold value of FECR < 95% to detect AR [34].

In the present study, AR was considered present if the FECR was below the threshold value in one or more of the above-mentioned methods.

In the EHA, the hatching percentage was calculated for each well (larvae/(eggs + larvae) × 100). The data was analysed in GraphPad Prism Version 5.1 (GraphPad Software, Inc., USA) to determine the concentration required to inhibit hatching of 50% of strongyle eggs (EC_50_), the 95% confidence interval, and the coefficient of determination (*R* ^2^). According to Coles et al. [9], resistance is declared when the calculated EC_50_ value is ≥0.1 μg/mL. Resistance in only a minor nematode species may be declared when EC_50_ < 0.1 μg TBZ/mL and concurrent hatching of larvae is seen in 0.3 μg TBZ/mL [52].

## Results

### Prevalences

**Figure 1.**
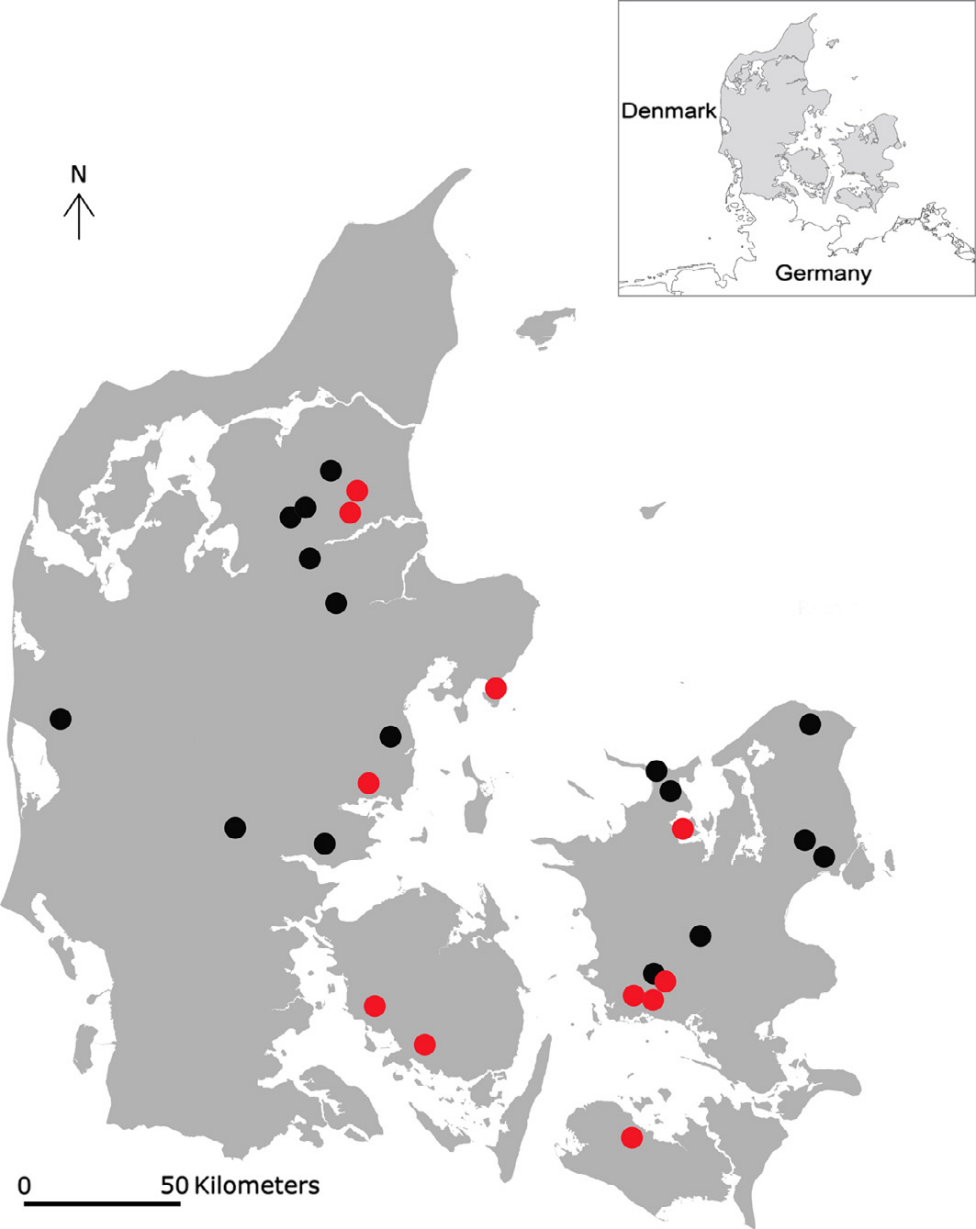
Geographical distribution of herds (*n* = 27) included in the prevalence study of gastrointestinal nematodes of Danish goats, 2012. Red and black dots: participating goat herds. Red dots: goat herds (*n* = 11) that tested positive for *Haemonchus contortus*.

Faecal samples were collected from 27 farms during the period 30 April–2 September 2012 (Fig. 1). In each herd 4–12 kids were sampled, totalling 252 individual samples. None of the goat owners reported clinical signs of parasitism among the sampled kids. The overall prevalence of nematode eggs was 77.0% and the herd prevalence was 89.0%. The corresponding overall prevalence of strongyle eggs was 69.0% and the herd prevalence was 81.5% (Table 1). *Nematodirus* spp. were found in 15.0% of the individual samples and 37.0% of the herds. *N. battus* was detected in four different herds (only identified in a single animal in each of three herds) before June. There were incidental findings of lungworm larvae (species not identified). A large variation was seen in the FECs, mainly due to the 10% samples in late season (Table 2). PNA staining was performed on samples from 12 farms and *H. contortus* was found in 11 out of these (Table 3).

**Table 1.** Prevalence of parasite eggs and oocysts in 252 goat kids from 27 Danish farms, 2012.

Parasite species	Herd prevalence (95% CI)	Individual prevalence (95% CI)
Strongyle eggs*	81.5 (63–92)	69.0 (63–74)
*Nematodirus battus*	14.8 (6–32)	3.6 (1–6)
Other *Nematodirus* spp.	37.0 (22–56)	15.0 (11–20)
*Strongyloides papillosus*	55.6 (37–72)	13.1 (9–17)
*Trichuris ovis*	63.0 (44–78)	33.7 (28–40)
*Capillaria longipes*	22.2 (12–41)	6.0 (3–9)
*Skrjabinema* spp.	18.5 (8–37)	4.0 (1–6)
*Moniezia expanza*	7.4 (2–23)	4.4 (2–7)
*Eimeria* spp.	100 (88–100)	99.6 (99–100)

95% CI: 95% confidence interval.

* Strongyle eggs including *Nematodirus* spp.

**Table 2.** Characteristics of the individual faecal egg counts (epg) from Danish goat kids in 2012.

Faecal strongyle egg count	Early summer (30/4–1/7) (*n* = 232)	Late summer (23/8–2/9) (*n* = 20)	Whole period (30/4–2/9) (*n* = 252)
Range	0–810	80–14,340	0–14,340
Mean (95% CI)	67 (51–83)	4213 (2598–5828)	396 (213–579)
SD	125	3540	1475
Median	15	3190	23

95% CI: 95% confidence interval.

**Table 3.** Detection of *Haemonchus contortus* eggs by Peanut Agglutinin Staining of pooled faecal samples from kids in 12 Danish goat herds, May–August 2012.

Farm number	Fluorescent eggs/total eggs counted	*H. contortus* (%)	Detection +/−
2	2/69	2.9	+
3	69/110	62.7	+
6	28/42	66.7	+
9	1/5	n.a.	+
10	32/108	29.6	+
16	86/109	78.9	+
18	90/100	90	+
22	2/83	2.4	+
23	0/29	0	−
25	1/106	0.9	+
26	108/116	93.1	+
27	1/107	0.9	+

n.a. = not applicable because of low numbers.

### Faecal egg count reduction test

In six herds no controls were included in the FECRT due to low numbers of kids, and thus only the McKenna method [34] for FECR was applicable (Table 4). AR against IVM was found in eight of the ten farms. In those farms where all three methods of calculation were applied (*n* = 4), agreement as regards IVM resistance was observed between the results of the different methods. Resistance against FBZ was present in all three farms tested (#2, 18 and 22), by one or more methods. In farm 2, resistance was only present according to Coles et al. [9], but the reduction percentage was close to the threshold value when using the two other methods. The mean FEC of control groups increased significantly in herd #22 and decreased in herd #2 (*p* < 0.05).

**Table 4.** Faecal egg count reduction percentages in 10 Danish goat herds in 2012, according to three different calculation methods.

Herd	Drug	*n*	Coles et al. 1992	Presidente 1985	McKenna (1990)
2	IVM	10	99.7 (97.3–100)	99.4	99.7
	FBZ	10	92.7 (84.9–96.5)^R^	90.8	95.3
	C	10	–	–	49.2
3	IVM	7	–	–	98.8
10	IVM	13	–	–	71.4^R^
16	IVM	13	–	–	65.9^R^
18	IVM	8	80.6 (62.2–90.1)^R^	82.8^R^	82.1^R^
	FEN	8	56.3 (27.7–73.3)^R^	51.1^R^	49.1^R^
	C	8	–	–	−4.0
22	IVM	10	84.5 (68–92.5)^R^	83.9^R^	49.7^R^
	FEN	10	21.2 (–157.5–75.9)^R^	−56.2^R^	−389.6^R^
	C	10	–	–	−213.5
23	IVM	10	−28.6 (−218.7–48.1)^R^	55.2^R^	45.9^R^
	C	10	–	–	−20.9
25	IVM	7	–	–	89.4^R^
26	IVM	10	–	–	84.1^R^
27	IVM	8	–	–	69^R^

R = AR is declared according to the specific calculation method. IVM = ivermectin, FBZ = fenbendazole, C = untreated controls. The 95% confidence interval is indicated in brackets.

### Egg hatch assay

By this method resistance to BZ was detected in all herds except #3 and #25 (Table 5). The three herds that tested positive for BZ resistance in the FECRT (#2, 18 and 22) were also positive in the EHA. The EC_50_ ranged from 0.069 to 9.803 × 10^6^ μg TBZ/mL, and the coefficients of determination (*R* ^2^) were all above 0.85. The hatching percentage in the negative controls was above 80% in all herds, except herd #18, in which the hatching percentage ranged between 41 and 51%. In herds #3 and #25, EC_50_ was below 0.1 μg TBZ/mL, but hatching of larvae was seen at a concentration of 0.3 μg/mL. When summarising the results from the FECRT and the EHA, seven of the ten herds had AR against both IVM and BZ in their nematode populations.

**Table 5.** Results from an egg hatch assay in ten Danish goat herds in 2012.

Herd	EC_50_ (μg TBZ/ml)	*R*^2^
2	0.11 (0.081–0.14)	0.95
3	0.087 (0.039–0.19)	0.87
10	~9.803 × 10^6^	0.91
16	0.21 (0.070–0.66)	0.94
18	0.11 (0.69–0.18)	0.87
22	~0.59 (very wide)	0.99
23	0.13 (0.084–0.19)	0.91
25	0.069 (0.050–0.095)	0.94
26	~28.52 (very wide)	0.99
27	~67,246	0.85

EC_50_ above 0.1 μg TBZ/ml indicates resistance against benzimidazoles. *R* ^2^ = correlation coefficient. The 95% confidence interval is indicated in brackets.

### Questionnaire

Questionnaire data were obtained from 27 of the 137 herds which were invited to participate in the study. Herd size ranged from 4 to 500 adult goats, with a mean of 55 goats and a median of 17 goats. Of the 27 herds included in the prevalence study of gastrointestinal nematodes and AR, 15 were hobby herds, of which two were registered as organic and four were zoological gardens. Among the 12 professional herds, 7 were organic, producing either milk, meat or both, and 5 were conventional, producing meat, milk or mohair fibres. Fifty-five percent of the herds had Boer goats, being the dominant breed, followed by “mixed breed” (44%).The three largest participating herds had a combination of milk-producing breeds (Saanen, Toggenburg and Danish Landrace). A total of 1129 kids were born in the kidding period from January to July with most kiddings during March–April in 19 of the herds (70%).

The average size of the professional herds in the study was 95 adult goats, whereas the hobby farms had an average size of 23 adult goats. No major differences regarding management were found between hobby and professional goat herds. However, all of the professional herds used pasture rotation, whereas this was only the case in 41% of the hobby farms. In addition, the professional herds treated more intensively with anthelmintics: 82% of the professional herds used whole-flock treatments compared with 50% of the hobby farms; and while hobby farmers treated 0.8 times per year in kids as well as adults, the professional farmers treated 1.2 and 1.0 times per year in kids and adults, respectively.

The mean number of treatments for kids and adult goats, respectively, was 0.96 and 0.89 treatments per year. Treatment frequencies are summarised in Table 6. Three herds (11%) never used anthelmintic drugs, 6 herds (22%) treated without any treatment plan, 7 (26%) treated only at signs of disease, and the remaining 11 (41%) had a predetermined treatment plan, e.g. to treat at kidding or turn-out. In 37% of the herds anthelmintic treatments were used selectively, whereas the remaining herds treated all animals.

**Table 6.** Number of anthelmintic treatments per year in 27 Danish goat herds, 2012.

No. of treatments	No. of herds (%)	
	Kids	Adult goats
0	9 (33)	10 (37)
1	11 (41)	10 (37)
2	6 (22)	7 (26)
3	1 (4)	0 (0)

Of the 24 herds that utilised anthelmintics, 15 (63%) used macrocyclic lactones at their last treatment, 4 (17%) used benzimidazoles, 2 (8%) used other drugs and 3 (13%) did not know which drug they had used. In addition, 15 (63%) herds had used the same drug repeatedly. As regards anthelmintic efficacy, only one farmer (4%) had noticed insufficient anthelmintic efficacy, 6 (25%) farmers did not know if the anthelmintic had any effect, and 17 (71%) farmers believed the anthelmintic treatment had the desired effect. All except one of the 27 herds in the study claimed to have a good understanding of AR.

Data concerning deworming of new animals prior to introduction into the flocks were obtained from 24 herds. Of these 12 (50% corresponding to 44% of the entire study population) introduced new animals in 2011; and 3 herds (25%) treated with anthelmintics before the introduction. Among the 12 herds which did not introduce new animals in 2011, 7 stated that they would use quarantine anthelmintic treatment prior to introduction of new goats.

As regards grazing practices, the goats were grazing in all herds except three zoological gardens where the animals only had access to dirt paddocks. Kids and adult goats were grazing together in 22 herds, while they grazed separately in one herd and the kids were kept indoors in the remaining herd. Of the 23 herds with grazing kids, pasture rotation was used in 16 (70%), and 12 (52%) had a specific grazing strategy including: rotating untreated goats to clean pasture (30%), “dose and move” (13%), co-grazing with other animal species (9%), strip grazing (4%) or “other strategies” (17%).

## Discussion

### Parasite fauna

The prevalences of gastrointestinal parasites found in the present study are similar to previous studies from Norway and Poland [14, 20], with the majority of kids having patent strongyle infection (69%, *n* = 252), and practically all kids (99.6%, *n* = 252) were infected with *Eimeria* spp.

Due to generally low FECs PNA was performed in less than half of the participating herds (*n* = 12). Had the samples been collected later the FECs would probably have been substantially higher, since egg excretion generally rises during the grazing season [6, 44]. *H. contortus* was found in 11 of the 12 tested herds (92%). Since only samples with high FECs were stained, the PNA results are subject to selection bias, and the detected herd prevalences may be falsely high. However, recent studies [5, 14] confirm a high prevalence even in northern temperate regions. In these areas *H. contortus* primarily over-winters as arrested larvae within the host [30]. Maturation of arrested larvae is expected to peak around kidding, which in this case was primarily in March–April.

Considering the prepatent period of around 2.5–3 weeks [12], it is assumed that the majority of potentially arrested *H. contortus* larvae should have matured and been transmitted to kids in May or later when the samples were collected. Therefore, animals infected with *H. contortus* would be expected to have a FEC above 300 epg at the time of sampling [42], and would consequently be detected in the present study. This means that the detection of *H. contortus* in 11 of the 27 herds (41%) most likely is a better estimate of the actual prevalence in the study population. Regardless of this, it is concluded that *H. contortus* is a widespread and well-established nematode species in Danish goats.

*N. battus* was only identified in 4 herds and 3.6% of all individual samples and diarrhoea was not reported by the owners, suggesting that *N. battus* overall is not a major pathogen in Danish goats. Since the majority of samples were collected during the peak period for *N. battus* egg excretion in Northern Europe [46], this prevalence is considered representative for Denmark.

### Anthelmintic resistance

Resistance against one or more anthelmintics was detected in 9 out of 10 herds (90%). In the FECRT, resistance against BZ was present in all of the three herds tested, and against IVM in eight herds (80%). Based on the EHA, resistance against BZ was found in 8 of the 10 herds (80%), and thus, dual resistance against both BZ and IVM was present in 7 herds (70%). A similar occurrence of AR was seen in Danish goat farms in 1996, where the efficacy of BZ, IVM and levamisol was tested using the FECRT and *in vitro* assays [32]. At that time AR against one or more anthelmintics was detected in 12 of 15 tested farms (80%); resistance against BZ was found in 10 out of 15 farms (67%), and against IVM in 2 out of 2 farms. Due to the low number of farms tested for AR against IVM in 1996, nothing can be concluded concerning the development of resistance against this group of drugs over time. Likewise, it is not possible to assess the national prevalence of AR, since only a few farms were included in both studies. Nevertheless, these studies indicate that AR is widespread and well-established among nematodes of Danish goats.

No anthelmintics are approved for use in goats in Denmark. Thus, IVM and FBZ were applied at 1½ and 2 times the dose levels recommended for sheep, which is common practice [1]. The subcutaneous route of administration was chosen for IVM as no oral formulation was registered for use in small ruminants in Denmark. The route of administration is known to significantly affect the pharmacokinetic behaviour of IVM [18, 19, 29]. However, reports concerning the significance of the administration route in goats are conflicting. Pearson and Rutherford [38] described reduced efficacy of IVM injection compared with oral administration at a dose of 0.2 mg/kg, in accordance with results reported by Gopal et al. [19]. However, more recent studies have shown that IVM plasma and tissue concentrations were significantly higher after subcutaneous injection compared with oral administration [18, 29]. Therefore, we cannot determine to what degree our results were affected by the route of administration.

In the present study the various methods used for calculation of FECR generally showed good agreement, and as expected, the largest differences were recorded in farms where there was a large increase or decrease in the FEC in the control group. A statistically significant difference in the FEC was recorded in control groups from herds #2 and #22. Most importantly, the methods agreed on the discrimination of resistance and susceptibility in all cases but one (#2). Regarding the EHA performed in this study, the hatching percentage in negative controls was high (>80%), and R_2_ was above 0.85 in all tests, indicating a good test performance [52]. BZ efficacy was tested in three farms with both the FECRT and EHA and full agreement was seen between the two tests in accordance with several previous studies. [11, 13, 32].

Mixed infections with both susceptible and resistant species may complicate interpretation of FECRT results, especially in cases with the FECR around the threshold level [8, 39]. Egg excretion can range from very high in some fecund species (such as *H. contortus*) to substantially lower in others, resulting in a higher or lower reduction percentage depending on species composition and resistance status. Pre and posttreatment larval cultures enable calculation of the specific FECR for each nematode species, thereby clarifying each species’ susceptibility level [9, 39]. In the present study 4 farms had a FECR in the range 80–95% and in these cases larval cultures would have been highly relevant. In the 7 herds with dual resistance, we cannot conclude if one or more species were resistant to both drugs, or if one was resistant to IVM and another to BZ. This also requires pre and posttreatment larval cultures.

Similarly, mixed infections can complicate interpretation of EHA results since varying susceptibility between species could result in a range of different EC_50_. This may explain the wide confidence intervals seen in the present study. Furthermore, the two herds (#3, 25) with EC_50_ below 0.1 μg TBZ/mL in the EHA had larval hatching at the discriminating dose (0.3 μg TBZ/mL). This indicates that resistant nematode strains were present in all of the ten samples, despite the calculated EC_50_ suggesting otherwise [52]. It has been suggested to lower the threshold for EC_50_ to 0.05 μg TBZ/mL if *H. contortus* is present in the sample [53]. Thus, as *H. contortus* eggs were detected in samples from 9 of 10 herds tested in the EHA a decreased EC_50_ threshold may have been more appropriate, and would have supported the detection of BZ-resistant strains.

### Questionnaire results

Goats are mainly held as large animal pets in Denmark, and 89% of Danish goat herds have less than 10 animals [26]. In the present study, a number of large, commercial dairy goat farms were included, and thus, only 56% of the herds were defined as hobby herds, resulting in a skewed herd size distribution compared with the true distribution. Accordingly, the focus of the present study was on larger goat herds in Denmark where good management (e.g. grazing strategies, quarantine treatment of introduced animals, strategies for anthelmintic treatment) is likely to be of significant importance for the farm economy and animal welfare. When comparing management risk factors associated with anthelmintic resistance between hobby herds and professional herds, only a few differences were observed: treatment intervals were slightly shorter and the whole flock was more widespread in the professional herds. Analysis of correlation between AR and management risk factors was not possible due to the low number of participating herds in the AR study. A larger sample size would have permitted such an analysis, but as the vast majority of goat herds in Denmark are small, this is difficult to achieve in practice.

Anthelmintic drugs were used in 89% of the herds, and 75% of these had a predetermined treatment plan. Examples of such plans were treatments at turn-out, kidding or housing. Treatment at signs of disease was the most common practice, and was used in 29% of the herds, not necessarily as a deliberate strategy but in several cases as an emergency solution. If selected individual animals are treated, e.g. at signs of disease, this is defined as a targeted selective treatment (TST) [23]. TST is an effective control measure to prevent AR, and despite the selective treatment, productivity in the untreated animals can be maintained [24, 49]. In the present study a total of 37% of the herds used TST: 50% of the hobby herds and 18% of the professional herds. This relatively restricted use of TST may have been influenced by limited awareness of the benefits of this strategy, fear of disease in untreated animals or the perception that TST is too laborious.

The mean number of treatments per year was 0.96 and 0.89 for kids and adult goats, respectively. In only 26% of the herds, kids and adults were treated with anthelmintics ≥ 2 times per year, and the maximum frequency was 3 treatments per year. This is markedly lower than observed in 1996 by Maingi et al. [32], who found that 70% kids and 90% adult goats were treated twice or more on a yearly basis, and more than 4 yearly treatments were seen in about 20% of all herds. This decline in treatment frequency is most likely associated with the introduction of the prescription-only legislation in Denmark in 1996. Generally, the Danish treatment frequencies are low compared with countries with high AR levels, such as South Africa [48], New Zealand and Australia [10, 27], and high treatment frequencies are probably not the major cause of AR in Denmark today. A further reduction of the treatment frequency, as recommended to limit AR development [2, 15], will be hard to implement under Danish circumstances, and is not considered realistic.

New animals were introduced into approximately 50% of the participating herds in 2011; and of these only 25% treated new animals with anthelmintics prior to introduction into the flocks. The lack of anthelmintic treatment and quarantine measures for goats that are moved between herds or imported from other countries as breeding animals facilitates the spread of nematode species, and is an important factor for dissemination of AR [41, 50, 54]. Considering the fact that treatment frequencies are fairly low, movement of host animals between flocks is likely to be a major reason for the widespread occurrence of AR in Denmark.

In conclusion, the present study demonstrated *H. contortus* in a large proportion of the tested goat herds; and widespread AR against the most commonly used anthelmintics at the dose levels recommended for goats, and management practices that are known to increase the risk of AR development. Efficiency of other anthelmintic classes, which were not tested in the present study, may still be sufficient against gastrointestinal nematodes in Danish goats. Nevertheless, there are several reasons for concern, and efforts to monitor and control further development of AR are warranted.
